# The evolution of ASEAN’s language policies: A diachronic analysis of official documents and website

**DOI:** 10.1371/journal.pone.0315076

**Published:** 2025-01-27

**Authors:** Chen Zhang, Ronghui Zhao, Yan Huang

**Affiliations:** 1 Center for Grenadian Studies, School of Foreign Languages, Ningbo University of Technology, Ningbo, Zhejiang, China; 2 China Center for Language Planning and Policy Studies, Shanghai International Studies University, Shanghai, China; 3 School of Foreign Languages, East China University of Science and Technology, Shanghai, China; Chulalongkorn University, THAILAND

## Abstract

Language policy plays a pivotal role in sustaining language behaviors and transforming language ideologies into practices. While the analysis of language policies in international organizations has received increasing attention, the evolution of language policies in the Association of Southeast Asian Nations (ASEAN) has been understudied. Existing research on ASEAN’s language policies has concentrated on its official language, often overlooking the language practices and ideologies embedded within these policies. This study seeks to investigate the evolution of ASEAN’s language policy by examining its official documents and website. We employed the *Wayback Machine* to trace the development of the ASEAN official website and adopted a corpus-based approach, utilizing tools such as AntConc and Sketch Engine, to analyze the Chairman’s Statements of the ASEAN Summit (CSAS). The study reveals three key insights: 1) The evolution of the ASEAN official website can be categorized into three distinct stages. 2) A consistent use of the collective pronoun “we” in the statements highlights the emphasis on cooperation and progress across economic and political-security domains. 3) The ASEAN community has persistently upheld its values, fostering an ASEAN identity and a genuine ASEAN community throughout its development. This diachronic analysis clarifies the characteristics of ASEAN’s language policy, deepening our understanding of the intricate relationship between language practices and ideology, and their implications for regional cooperation and integration.

## Introduction

The Association of Southeast Asian Nations (ASEAN), established on August 8, 1967, in Bangkok by the founding members—Indonesia, Malaysia, the Philippines, Singapore, and Thailand—has experienced significant growth and expansion over the years. Joining the original quintet were Brunei Darussalam on January 8, 1984, Vietnam on July 28, 1995, and Laos and Myanmar on July 23, 1997, followed by Cambodia on April 30, 1999. The formation of the ASEAN Community on December 31, 2015, marked a pivotal moment, signifying the completion of the regional integration process within the three core pillars of political-security, economy, and socio-culture. This evolution has solidified ASEAN’s conceptual framework and values in regional and international relations, establishing a robust institutional framework that underscores the region’s importance on the global stage in Southeast Asia [1].

At the apex of ASEAN’s decision-making hierarchy lies the ASEAN Summit, whose principal document, the Chairman’s Statement, encapsulates the achievements and anticipated outcomes of ASEAN’s tenure. Since its inception, Article 34 of the *ASEAN Charter* has designated English as the working language, affirming its status as the lingua franca [2]. This monolingual policy is evident on ASEAN’s official website (asean.org), where all content is presented in English, serving as a key element of the ‘Virtual Language Landscape’ (VLL) [3] and a dynamic tool for language planning. The website, which Hine [4] refers to as a ‘repository of texts,’ encompasses an extensive collection of documents and news updates, providing a rich resource for the study of ASEAN’s language policies. Official statements from institutional authorities are of particular interest, as they often utilize language to illuminate their motivations and beliefs [5]. The website is thus well-suited for examining ‘the degree of compliance with relevant local language legislation or de facto policy in cases where no legislation exists’ and the role of English [6, p. 130]. Recently, Lee et al. [7–9] have explored the manifestations of English as the sole language policy from various perspectives, including language ideology, the ASEAN Way, and functional identity, thereby elucidating various forms of language practices. However, systemic examination of language policies and language practices via official websites, particularly employing a corpus-based methodology to examine the Chairman’s Statement from the ASEAN Summit (hereinafter referred to as CSAS), remains comparatively unexplored.

This study draws on the data from ASEAN website, accessed via the *Wayback Machine*, to trace the trajectory of language practices evolution up to January 2024. By focusing on the corpus-based analysis of the CSAS from 1976 to 2023, this research aims to reveal the underlying ideologies that shape ASEAN’s language practice and to illuminate how these policies have evolved over time to reflect the organization’s maturation and its dedication to regional cooperation and integration.

This article is organized into several sections. The first section provides a summary of previous studies on ASEAN’s language policy and language practice. It critically examines existing studies, highlighting gaps in the literature and potential areas for further exploration. The second section outlines the research methodology utilized in this study. It details the data collection and analysis processes used to achieve the specific objectives. The third section presents the findings of the study, supported by rigorous data analysis. The final section offers a conclusion, discusses limitations, and suggest potential directions for future research.

## Literature review

### Language policy and language ideology

Spolsky [10, p. 5] posits that language policy comprises three interrelated yet independent components: language practice, language beliefs and values, and language management. Language practice denotes the discourse articulated by speakers for specific purposes; language beliefs and values are significantly influenced by language practice and, in certain instances, evolve into language ideologies. Language management is enacted by individuals or entities wielding linguistic authority, and to a certain extent, it reflects the language ideology of those in positions of authority. Thus, within this framework, policy documents can be construed as manifestations of language practices executed by authorities possessing a discernible level of language ideology. Simultaneously, the layout of the official website and the documents it presents are also significant expressions of language practices. Zhang et al. [11] explored language choices on International Organizations’ official websites and focused on the roles of these websites.

Language policy is inherently shaped by ideological perspectives and manifests these ideologies throughout the policy formulation process. Beneath the veneer of policy documents lies the language policy embedded within the text itself, which serves to regulate ideology by structuring the text and determining its linguistic form [5]. When discourse practices within policy texts either uphold or challenge power dynamics, they acquire ideological implications [12].

### Language policy in international organizations

Numerous scholars have directed their attention towards delineating the characteristics of language policy within international organizations. They have delineated the language features inherent to these organizations, scrutinized the impact of language ideology, investigated language practices across various tiers of international bodies, and delved into the multitude of factors influencing language policy and planning within such organizations [13, 14].

In the context of ASEAN, language education policies have garnered particular interest, with researchers assessing the impact of these policies on the preservation of linguistic and cultural diversity and the promotion of both English and the member states’ national languages [15, 16]. The ASEAN community has been scrutinized as a regional case study to understand its linguistic landscape [2], with efforts concentrated on achieving a harmonious equilibrium among the official language, domestic languages, and local dialects of ASEAN [17]. There has also been a push to innovate and enhance multilingual education strategies within the region [15, 18]. Additionally, discourse on “ASEAN identity” has gained momentum, often analyzed through the lens of the principles of non-interference and consensus-driven decision-making that define the “ASEAN Way,” as the region navigates its future direction [19, 20].

Currently, a significant point of contention is the monolingual ideology that underlies ASEAN’s language policies. Low and Ao [21] offer a comprehensive overview of ASEAN’s English policy, the spread of English, and the balance it strikes within the region. Some argue that English serves primarily to enhance ASEAN’s ‘functional identity,’ falling short of deeply embedding a ‘socio-cultural identity’ [9] and is often viewed as a ‘neutral’ tool for communication [7]. In light of these discussions, there is a growing advocacy for moving beyond the monolingual framework towards an ‘ecology-of-languages’ approach, which is more inclusive and sustainable for regional language policy [8].

### Discourse analysis and corpus linguistics in international organizations

Discourse analysis entails a systematic examination of the interplay between discourse, agents, and the construction of social reality [22]. This analytical approach encompasses a broad spectrum of discourse materials, including non-verbal signals, visual imagery, symbolic representations, and both written and spoken language [23]. In recent years, corpus linguistics (CL) has emerged as an innovative analytical tool that complements traditional discourse analysis. As a computer-assisted method for text analysis, CL is recognized for enhancing the rigor and reliability of findings by providing a more objective lens through which to examine language use [24].

Corpus linguistics equips researchers with the ability to identify salient linguistic patterns, themes, viewpoints, and attitudes that may otherwise remain obscure within vast sets of unstructured data. By integrating corpus linguistics with language policy discourse analysis, scholars have been able to trace the evolution of language-related issues and priorities in speeches by United Nations Secretary Generals and Member States over a period of 46 years [13]. Similarly, Brett and Marika [5] applied a corpus-driven analysis to legislative documents from the U.S. Congress, revealing the semantic preferences and ideological underpinnings within the collocational patterns of language policy.

Considering these insights, the application of corpus linguistics to discourse within international organizations is both viable and essential for uncovering the deeper layers of consciousness and values that shape organizational discourse. While ASEAN’s developmental concepts and values have been explored in the context of diplomatic relations [1] and political science [25], there is a noticeable gap in multimodal analyses that leverage the content and evolution of official websites. Specifically, there has been a lack of focus on how website changes and the application of corpus linguistics methods to the analysis of Chairman’s Statements (CSAS) might shed light on the developmental ethos of ASEAN. To address this gap, this study poses three interrelated questions:

**RQ1.** What are the phased characteristics of the historical archive versions of the. ASEAN official website from 2006 to 2024?

**RQ2.** What development priorities of ASEAN are reflected in the CSAS?

**RQ3.** What language ideologies are embedded within these characteristics and priorities?

## Methodology

### Data collection

The ASEAN’s official website (asean.org) serves as the central repository for information, meticulously documenting the site’s evolution and encapsulating the discourse found within the official statements it hosts. To systematically extract relevant documents, we utilized the Advanced Search Tool, focusing on the keyword “ASEAN summit”, which yielded a preliminary collection of 429 statements. Following a rigorous screening process, we curated a targeted corpus and compiled a dataset consisting of Chairman’s Statements of the ASEAN Summit (CSAS). Our primary dataset, the “CSAS” corpus, covers an extensive timeframe, spanning 47 years from 1976 to 2023. The first ASEAN summit, held in February 1976 in Bali, Indonesia, was a seminal event where the Treaty of Amity and Cooperation in Southeast Asia (TAC) was signed, and the Bali Declaration underscored the importance of coordination and unity among ASEAN nations. This historic summit marks the starting point of our corpus. As of January 2024, we identified and accessed 40 publicly available statements on the ASEAN site. To facilitate thematic comparison and contextual analysis within the framework of this international organization, we incorporated a parallel corpus of American English discourse. This comparative dataset was integrated into AntConc 4.2.4, a cutting-edge tool for corpus linguistic analysis. Table 1 provides a detailed breakdown of the document counts within both corpora, establishing a structured foundation for our comparative analysis.

**Table 1:** Research corpus.

Database	Corpus	Years	Types	File Tokens
**Research corpus**	CSAS	1976–2015	6,609	126,016
		2016–2019	4,788	58,781
		2020–2023	4,816	72,916
		Total	8,981	257,713
**Parallel corpus**	American English	2006	52,456	1,017,879

### Data analysis

Our initial analysis involved a meticulous examination of the distribution and content evolution of the official ASEAN website across three distinct phases, facilitated by the *Wayback Machine*. This was followed by a comprehensive corpus-based analysis of the CSAS statements to identify ASEAN’s developmental priorities throughout these phases.

Firstly, in order to trace the development of language policy on this website, this study adopts a diachronic approach in line with the perspective of Kelly-Holmes [6, p. 135], which advocates for “recording changes in language provision and content over time to track the emergence of language policies.” The *Wayback Machine* (https://archive.org/web/), an invaluable tool since its launch in 2001, has been instrumental in this process, capturing and archiving billions of web pages and multimedia files from across the internet over the past two decades. This non-profit digital library serves as a critical resource for researchers, enabling the collection of diachronic data from websites and facilitating the retrieval of historical website content. It offers a valuable large-scale data source for analyzing web information over time, providing a comprehensive view of how online content evolves [26].

Secondly, we adopted a corpus-based discourse analysis approach, which blends the quantitative rigor of corpus linguistics with the qualitative depth of discourse analysis [27, 28]. This methodology enables researchers to investigate patterns of ideological consistency and transformation within textual outputs over time [29]. By identifying statistically significant word frequency patterns, we can uncover and scrutinize the language ideologies embedded within the corpus [30]. In order to map ASEAN’s developmental trajectory, we employed AntConc 4.2.4 [31] for keyword, concordance, and Keyword in Context (KWIC) analyses, thereby determining the prevalence of topics and semantic inclinations. Additionally, we also utilized *Sketch Engine*, an online text analysis tool (https://www.sketchengine.eu/) [32], known for its intuitive visual effects that facilitate the generation of word frequency distribution maps, enhancing the comprehension of data and analysis outcomes. This multifaceted analytical approach ensures a nuanced and robust interpretation of the data, shedding light on the evolution of ASEAN’s language policy and its underlying ideologies.

## Results

### Evolution of ASEAN’s official website

Utilizing the W*ayback Machine*, we are able to virtually travel back in time and experience the ASEAN official website as it appeared in years past. Specifically, we accessed the official ASEAN website using the W*ayback Machine* on April 1, 2024. This tool allows us to not only view but also navigate through the website’s archived versions, providing a comprehensive understanding of how the distribution of web pages has changed over time.

#### The snapshot changes

**Fig 1:**
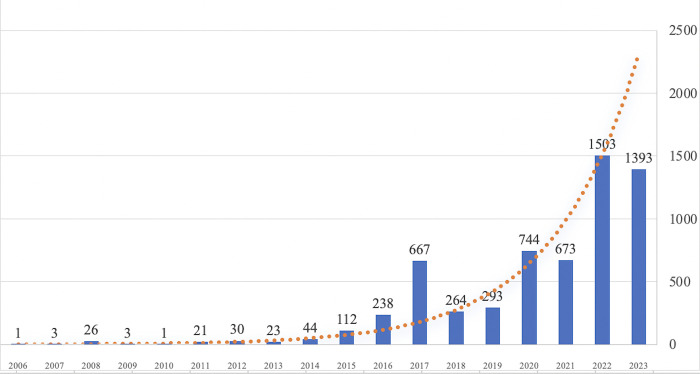
ASEAN website snapshots by year (2006–2023). Note: This figure illustrates the frequency of website snapshots taken each year, with the horizontal axis representing the year and the vertical axis representing the total number of snapshots captured annually.

The *Wayback Machine*’s earliest archived snapshot of the ASEAN website dates back to 2006, marking the starting point for our analysis. Over the years, the website has undergone a discernible evolution in its content update patterns (See Fig 1). Prior to 2011, updates were sporadic and infrequent. However, a notable shift occurred after 2011 with the website adopting a more consistent and intentional approach to regular updates. This trend became particularly pronounced after 2015, where the website’s maintenance and revision schedule became not only more frequent but also more stable and predictable. This evolution in the website’s development is a key indicator of ASEAN’s increasing awareness of the significance of digital communication shaping its identity and facilitating its regional objectives. Fig 1 illustrates the number of times the website’s pages have been snapped from 2006 to the end of 2023.

#### The layout and design changes

Beyond the specific content updates, the layout and design of the ASEAN official website also serve as a mirror to its phased development characteristics. To meticulously assess the official focus and the frequency of modifications to the website, this analysis will delve into the distribution changes and phased features of the ASEAN official website’s design across three distinct stages.

*1*. *Stage 1*: *Initiation (1976–2015)*. Prior to 2008, the design of the ASEAN website was relatively rudimentary, lacking significant branding or slogans that encapsulated the essence of the ASEAN community. The site’s appearance was disjointed, not yet coalescing into a cohesive representation of a regional organization. This initial stage precedes the formal establishment of the ASEAN Community in 2015, during which the foundational elements of community formation were laid.

*2*. *Stage 2*: *Expansion (2016–2019)*. The landscape of the ASEAN community’s online presence began to shift significantly in December 2015, marking a pivotal moment in its evolution. The website introduced a new vision, “ASEAN Community”, alongside a rallying motto, “One vision, one identity, one community”, and a shared vision, “ASEAN: A Community of Opportunities”, which encompassed three core pillars: Political-Security Community, Economic Community, and Socio-Cultural Community. From the end of December 2015 to 2020, the consistent positioning of the motto and shared vision on the homepage signified a period of relative stability in ASEAN’s developmental trajectory. This second stage, spanning from 2015 to 2019, was characterized by a focus on consolidating stability and fostering development within the community.

*3*. *Stage 3*: *Consolidation (2020-Present)*. On March 6, 2020, a subtle yet significant shift occurred as the shared vision of the three pillars was refined to “ASEAN: A Community of Opportunities for All”, underscoring a commitment to inclusive development across all member states and the broader region. Further changes emerged on August 7, 2021, when the website’s homepage was restructured, prominently displaying “ASEAN: A Community of Opportunities for All” adjacent to the ASEAN Community emblem. Concurrently, the former motto was retired, and the common vision was updated to “ASEAN Community Vision 2025: Integrating Countries, Integrating Development”, heralding a refreshed vision for the future. This third and current stage, which commenced in 2020, continues to the present, highlighting the dawn of a new era with a renewed emphasis on inclusive and integrated development.

In sum, by examining the evolution of the ASEAN website’s layout, we have delineated the development of the ASEAN community into three distinct stages, characterized by significant alterations in the presentation and content of its name, motto, and shared visions. These deliberate design choices and updates not only reflect the ASEAN community’s growing maturity and confidence but also its adaptive approach to regional integration and its commitment to fostering a unified and collaborative identity among its member states. The meticulous documentation and analysis of these changes provide valuable insights into the strategic communication and the evolving language policy of ASEAN, as it navigates the complexities of regional cooperation and identity formation in the 21st century.

### Corpus analysis of the “CSAS” document

Since 1976, a total of forty statements on diverse themes have been compiled (See Table 2). These will be analyzed through keyword, concordance, and KWIC methods.

**Table 2:** Themes and published years of CSAS.

Stages	Summit range	Time range	Documents number
**1**	From *The Declaration of ASEAN Concord* to *Chairman’s Statement of the 27*^*th*^ *ASEAN Summit*	1976.2.24–2015.12.21	27
**2**	From *Chairman’s Statement of the 28*^*th*^ *and 29*^*th*^ *ASEAN Summits* to *Chairman’s Statement of the 35*^*th*^ *ASEAN Summit*: *advancing partnership for sustainability*	2016.9.7–2019.11.3	7
**3**	From *Chairman’s Statement of the 36*^*th*^ *ASEAN Summit* to *Chairman’s Statement of the 43*^*rd*^ *ASEAN Summit*	2020.6.26–2023.9.5	6

#### Keywords across 3 stages

**Fig 2:**
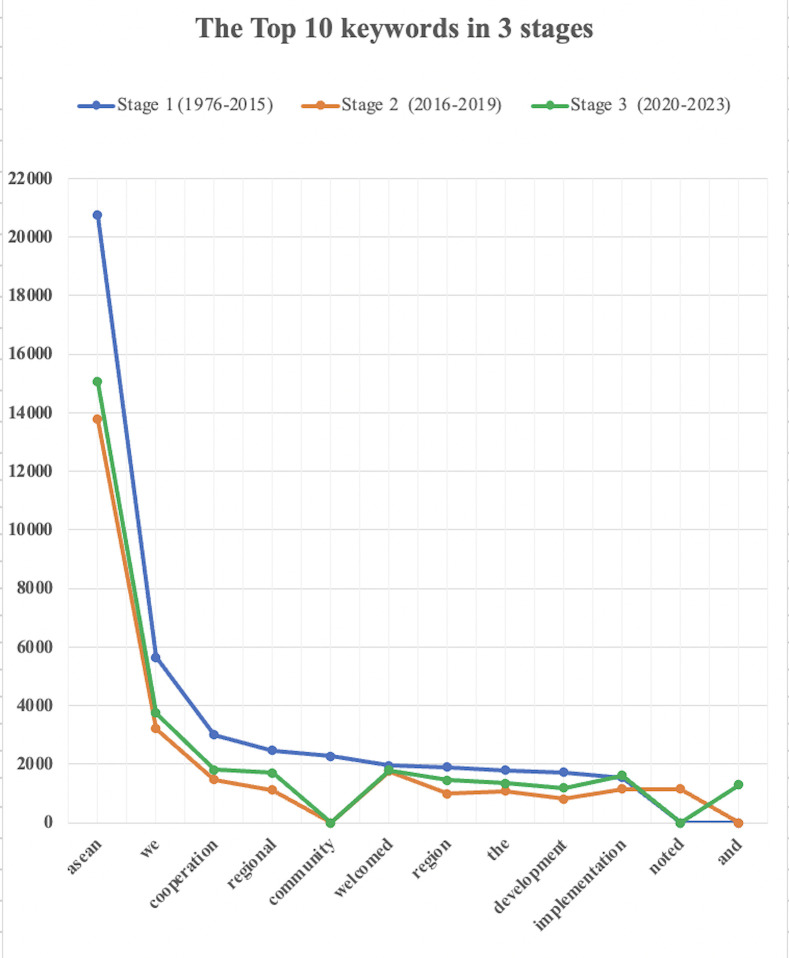
Frequency of top 10 keywords across three stages of ASEAN website development. Note: The horizontal axis represents the top 10 keywords identified across the three developmental stages of the ASEAN website. The vertical axis indicates the frequency of these keywords’ occurrences. The three lines depict the trend of keyword frequency over time.

Keywords are terms that appear with significantly higher frequency in a specific corpus compared to a reference corpus [33, p. 55]. These words are indicative of the core themes that are consistently emphasized across all texts within the database. The first 10 keywords (See Fig 2) in the content of the CSAS during these three periods are generally the same, reflecting the consistent development goals of the ASEAN community. There are some common characteristics:

*1*. *Prioritization of “ASEAN” and “We”*. Despite the establishment of a new ASEAN community at the end of 2015, the name representing this regional organization has remained unchanged. “ASEAN” and “we”, consistently rank as the foremost discourse elements, signifying the organization’s identity and agency. The release of Chairman’s Statement of ASEAN Summit over the years has announced its constant development focus and goals, emphasizing the functional symbol.

*2*. *Emphasis on “Cooperation”*, *“Regional”*, *“Development”*, *and “Implementation”*. According to the Bangkok Declaration of August 8, 1967, the initial goal of ASEAN was mainly to accelerate economic growth, social progress, and cultural development in the region through joint efforts, grounded in the principles of equality and partnership. This was aimed at establishing a prosperous and peaceful community of Southeast Asian nations, while also promoting cooperation and development in other areas. Therefore, it is not surprising that items such as “cooperation”, “region”, “development”, and “implementation” are consistently highlighted as areas of focus.

*3*. *Variations in development priorities and goals across periods*. Before the ASEAN Community was officially established by the end of December 2015, the term “community” itself was notably absent in later stages of discourse. However, through clustering, phrases, such as “ASEAN community” (338), “community building” (119), “community we” (107), “economic community” (70), “security community” (64), and “cultural community” (47), were frequently mentioned, with the numbers in parentheses indicating frequency. This indicates that the establishment and construction of the ASEAN community at this stage is the core task, aiming to establish a new community with “economy”, “political-security”, and “culture” as the three pillars.

In the second stage, during the early years of the establishment of the new ASEAN community, the term “welcomed” emerged as a key word, indicating a positive stance in fostering the development of ASEAN regional organizations. This word reflects an extremely welcoming and open approach towards embracing and accommodating the development and cooperation among member countries, while also encouraging a healthy competitive spirit. Through clustering, we found that the phrase “we also received” (54), “received the option” (28), “received the progress” (19), “received the signing” (16), “received the convention” (12), “received the establishment” (12), “region we received” (11), “received the successful” (10), “regard we received”(10), and “welcomed the completion” (8) were frequently used, reflecting the early establishment of ASEAN and the image it projected to the outside world.

In the third stage, its focus shifts towards the common regional development of member states, while also respecting the individuality of each state and enhancing connections with other international organizations. Through cluster analysis of the term “region”, regional development primarily centers on phrases such as “of the ASEAN region” (22), “regional plan of action” (14), “regional peace and stability” (9), “regional and international issues” (6), “regional and international organizations” (6), “regional comprehensive economic partnership” (6), “regional forum ARF and” (6), and “foundation for maintaining the region” (6). By maintaining the stability and development of its member states and providing more development opportunities and goals, the ASEAN community aims to achieve synchronized development and to solidify its position on the global stage.

#### Concordance analysis

Concordance (keyword-in-context analysis) is the second approach employed in this study. Concordance is essentially a qualitative method and involves a drilling down process. It allows for the comprehensive review of a keyword’s occurrences throughout the entire corpus. By facilitating a more granular analysis of corpus data, Concordance Analysis enables researchers to uncover emerging themes and patterns that manual coding might overlook. This methodological rigor is crucial for a comprehensive understanding of the data, as it reveals the intricate relationships and contextual uses of keywords within the corpus [34].

An in-depth concordance analysis was subsequently conducted on the first two keywords with the highest keyness values to elucidate their distinct characteristics and to uncover unique patterns of usage within the corpus. This analysis is crucial for understanding the consistent development philosophy and objectives that ASEAN upholds. The focus of this section is to analyze the combination of several keywords, thereby enhancing our understanding of the operation of ASEAN regional organization. Table 3 shows the top 20 concordance of the term “we” and Table 4 shows the top 30 concordance of the term “cooperation”.

**Table 3:** Top 20 concordance of the term “we”.

Rank	Collocate	Frequency R	Likelihood
**1**	welcomed	832	4966.246
**2**	Noted	622	3689.062
**3**	Also	591	3603.969
**4**	reaffirmed	374	2267.131
**5**	looked	355	1829.245
**6**	reiterated	213	1326.545
**7**	commended	208	1263.552
**8**	encouraged	213	986.637
**9**	recognised	161	940.002
**10**	agreed	175	813.822
**11**	acknowledged	131	806.988
**12**	underscored	130	793.818
**13**	expressed	134	723.173
**14**	tasked	118	676.256
**15**	stressed	95	578.308
**16**	appreciated	81	468.583
**17**	recognized	70	417.571
**18**	welcome	68	372.596
**19**	adopted	83	354.638
**20**	urged	64	350.516

**Table 4:** Top 30 concordance of the term “cooperation”.

Rank	Collocate	Frequency Left	Likelihood
**1**	regional	85	282.172
**2**	economic	75	277.408
**3**	apt	32	229.955
**4**	functional	16	154.815
**5**	practical	23	141.474
**6**	maritime	26	130.184
**7**	closer	17	98.667
**8**	enhance	28	95.641
**9**	strengthening	26	92.119
**10**	energy	23	91.392
**11**	cybersecurity	17	90.967
**12**	strengthen	25	82.180
**13**	defence	17	79.287
**14**	security	29	68.238
**15**	development	37	66.581
**16**	three	19	66.059
**17**	narcotics	7	55.291
**18**	enhancing	16	54.850
**19**	gulf	5	52.258
**20**	deepen	10	48.079
**21**	minerals	8	46.808
**22**	judicial	6	44.844
**23**	intensify	10	44.513
**24**	stronger	8	40.114
**25**	asean	99	34.152
**26**	shanghai	3	31.350
**27**	improving	7	30.278
**28**	industrial	7	30.047
**29**	enhanced	8	29.340
**30**	cultural	10	27.840

Utilizing Concordance Analysis, we have established a threshold for keyword frequency, requiring that terms appear at least 10 times within the corpus. Among the top 50 collocates of the term “we”, we have identified two primary categories. The first category emphasizes the concept of identity or the collective as the behavioral subject. For example, “We, the Heads of State/Governance of ASEAN Member States” appears a total of 35 times across the three stages of development. The distribution is as follows: the first stage with the highest frequency of 21 occurrences, the second stage with 8, and the third stage with 6. This pattern indirectly reflects that in the early stages of ASEAN’s establishment and development, the community highlighted its position and role as a leader, conveying a symbol of identity to the people. As development goals evolve, the collective or leadership identity role diminishes, gradually shifting focus towards the individual development of member countries. The second category is a type of behavioral subject that reflects the positive, inclusive, and accepting attitude of leaders towards the development of ASEAN. These words mainly include “welcomed”, “noted”, “realized,” etc.

The term “cooperation” refers to a collaborative endeavor that extends across diverse fields, institutions, and nations, as well as the attitudes that accompany such partnerships. Firstly, the focus lies on the areas of cooperation, including economics, marine affairs, energy, cybersecurity, minerals, etc., which are pivotal industries and fields pertinent to the development of regional organizations. Secondly, it centers on collaborating countries such as APT, namely ASEAN Plus Three, which includes the People’s Republic of China, Japan, and the Republic of Korea. Thirdly, this underscores the cooperative stance, with the objectives of enhancing, strengthening, and defending these partnerships, thereby fostering closer, stronger, and deeper cooperative relationships.

#### KWIC

**Fig 3:**
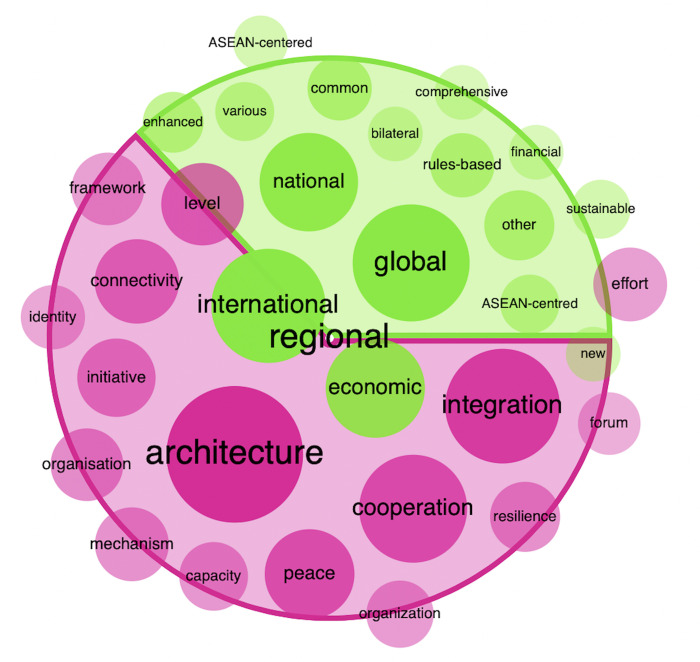
Word sketch of the term “regional” in CSAS. Note: This visualization is generated using the Sketch Engine tool. It displays the 16 strongest lexical combinations associated with the term “regional”. Red circles represent nouns that are modified by “regional”, while green circles denote the lexical combinations “regional” and/or…. The size of each circle is proportional to the strength of its association with “regional”, with larger circles indicating a stronger relationship.

We also performed a Keyword-in-Context (KWIC) analysis to delve deeper into the corpus and enrich our qualitative and contextual understanding. We selected the term “regional” as a case study to demonstrate our approach. This term serves as a lens through which we can examine the nuances of language use within the corpus. The KWIC analysis of “regional” provides a detailed view of the term’s occurrences, allowing us to explore its usage in context and understand its significance within the discourse. To further vivify our findings and elucidate the term’s relationships with other concepts, we present a network map to show the semantic networks associated with “regional” (see Fig 3), revealing how it is intertwined with discussions of regional cooperation, integration, and identity within the ASEAN framework.

Recognizing the vital role that robust regional development plays in the overall advancement of the ASEAN community, a series of efforts are being implemented within the region to promote cooperation. These initiatives include:

1. Mainstreaming the rights of persons with disabilities, enhancing digital transformation, ensuring food security and nutrition during crises, fostering greater regional collaboration to protect, preserve, and conserve cultural heritage, which forms the foundation of ASEAN’s regional identity, and addressing Social Service Workforce to Population Ratios.

*“We recognized the importance of ensuring that sub-regional development strategies are aligned with the overall development strategies of ASEAN”*. (From Chairman’s Statement of the 37^th^ ASEAN Summit “Cohesive and Responsive”, 2020).

2. Strengthening attention to women, children, adolescents, and the elderly through numerous documents and targeted step-by-step measures. For instance, it adopted the *ASEAN Leaders’ Declaration on Gender Equality and Family Development*, as well as the *Regional Plan of Action on the Elimination of Violence Against Women* (RPA on EVAW) and the *Regional Plan of Action on the Elimination of Violence Against Children* (RPA on EVAC) through relevant impact initiatives. Additionally, it facilitated the rollout of the *Regional Plan of Action on Implementing the Kuala Lumpur Declaration on Aging*: *Empowering Old Persons in ASEAN*, which aims to accelerate ASEAN’s efforts to address the challenges of demographic change and ensure inclusive and sustainable development. Furthermore, it reiterated the need to elevate the roles of children and youth in achieving sustainable development in the region, ensuring that they are prepared and future-ready to respond to complex global challenges through initiatives such as the *ASEAN Youth Academy Program*, *ASEAN Youth on Climate Change*, and *ASEAN Walk 2022*. This includes the *Regional Plan of Action on Implementing the ASEAN Declaration on the Rights of Children in the Context of Migration*, *Regional Plan of Action for the Protection of Children from All Forms of Online Exploitation and Abuse in ASEAN*, and the *Report on the 5th Domain of ASEAN Youth Development Index*.

3. A series of documents and implementation have been introduced to cope with natural disasters, such as the *ASEAN Agreement on Disaster Management and Emergency Response (AADMER)* Work Program 2021–2025, which aligns with the *ASEAN Vision 2025 on Disaster Management*. Besides, ASEAN strengthens its connections and cooperation with other international organizations, such as the United Nations, and is committed to two main goals: 1) leveraging the support of international organizations to develop key areas such as mental health, the tourism industry, a culture of peace, modernization, and the marine sector; 2) being well-prepared to engage in collaborative activities with international organizations, particularly in combating epidemics and drug-related issues. ASEAN is also committed to adhering to international laws and regulations, as emphasized in the Chairman’s Statement of the 40^th^ and 41^st^ ASEAN Summit (2022):

*“We also highlighted the importance of and reaffirmed our commitment to further strengthening ASEAN’s relations with other external partners as well as regional and international organisations, including the UN to address global concerns, to promote an enabling environment for peace, stability and prosperous development, to pursue shared goals and complementary initiatives, and to promote sustainable development for the benefit of our people*”.

Last but not least, ASEAN is maintaining an ASEAN-centered regional architecture that is open, inclusive, transparent, and rules-based.

*“We reiterated the importance of maintaining an ASEAN-centered, open, inclusive, transparent, resilient regional architecture that upholds international law and rules that strengthen our engagement and cooperation with Dialogue Partners and external partners, including through existing ASEAN-led mechanisms, to promote peace, stability, security, and development.”* (From Chairman’s Statement of the 43^rd^ ASEAN Summit, 2023)

## Discussion

This article has employed the *Wayback Machine* tool and corpus-based approach to explore the ASEAN’s language policies and practices, utilizing the ASEAN official website as the primary data resource. Drawing insights from the evolution observed across three distinct stages of the website, coupled with an examination of the thematic content and operational priorities articulated in Chairman’s Statements, the ensuing conclusions are synthesized.

Firstly, we identified three distinct evolutionary stages of the ASEAN official website with its symbolic and informational functions, highlighting the construction of an ASEAN community, the emphasis on cooperation, and the pursuit of regional integration. These delineated stages align seamlessly with ASEAN’s overarching development philosophy and ideology. At the establishment of ASEAN Community, the aims and purposes were on the one hand to bring about cooperation in the economic, social, cultural, technical, educational and other fields, and on the other hand, in the promotion of regional peace and stability through abiding respect for justice and the rule of law and adherence to the principles of the United Nations Charter. Ricento [35] once summarized three types of factors which were instrumental in shaping language policy, macro sociopolitical, epistemological and strategic. The establishment and goals of ASEAN were primarily concerned with political factors, including the impact of the Cold War, followed by economic and international affairs, both domestically and abroad. Initially, the five ASEAN pioneer countries pursued primarily political goals, aiming for peace and security in the Southeast Asian region. However, in 1992, ASEAN decided to establish the ASEAN Free Trade Area [36] and adopted ASEAN Vision 2020, a long-term road map guiding member states to the year 2020, and ASEAN Community Vision 2025. These initiatives were designed to gain more steam for the sake of navigating through a period of strategic and political challenges, economic stagnation, and concerns arising from major power competitions and the ongoing pandemic outbreak. They sought to earn increased respect from member states and dialogue partners to ensure ASEAN’s continued role as a key catalyst for peace, stability, and prosperity in Southeast Asia and beyond [37].

Secondly, we observed ASEAN’s unwavering focus on the collective “we,” underscoring the importance of collaboration and development across economic and political-security spheres, making an inclusive regional organization have always been the consistent principles of development values of ASEAN community. When ASEAN was formed in 1967, it was mainly seen to be a bulwark against the spread of communism [38] and this was what united the five founding nations-Indonesia, Malaysia, Philippines, Thailand and Singapore, Notwithstanding the Blueprints for achieving the APSC, the ASEAN initiatives of regional security dialogues through the ASEAN Regional Forum (since 1994) and the ASEAN Defence Ministers’ Meeting Plus 3 have successfully evolved into platforms for policy coordination [39, p, 11]. All these could be the safeguard for their economy which holds the first role in the regional process. The establishment of the ASEAN Economic Community (AEC) on December 31, 2015, aimed to reduce the development disparity among its member states and transition the region into a stable and prosperous community. This initiative envisaged a tightly integrated and cohesive regional economy aimed at fostering sustained economic growth. Through the *Bali Declaration of Agreement II* in 2003, the heads of government of all ASEAN member countries agreed on the vision of transforming ASEAN into a “One Vision, One Identity, One Community” society by 2025. This represented a transition from state-oriented to people-centered, and from a state-based organization to a community-based organization, under the theme “A Community of Opportunities for All” [40].

Thirdly, our analysis showed that the ASEAN community has consistently upheld its common values throughout its development, fostering a sense of unity and ultimately constructing an ASEAN identity and a genuine ASEAN community. The third pillar of the ASEAN Community is the ASEAN Socio-Cultural Community (ASCC). Maramis [41, p. 179] calls the ASCC ‘by a wide measure the most adaptive, reengineered, and reinvented pillar of the ASEAN Community.’ To promote better quality of life for the peoples and their communities in ASEAN, the ASCC aims at cooperation between the member states in areas such as culture and information, education, sports, social welfare and development, but also labour, women and gender, environment, poverty eradication, disaster management, and science and technology. With such a varied area to cover, it will come as no surprise that the community building process has often baffled both researchers and the general public, giving rise to a large number of initiatives from study days and conferences to action plans and reports. Standing on fundamental premises and taking a long-term perspective, one would say that the core of the ASEAN Community is the Socio-Cultural Community conceived as a vehicle for developing a sense of Southeast Asian identity, building a regional awareness and fostering mutual understanding among the people of ASEAN [38]. The ASCC Blueprint 2025 is another blueprint following the previous one, which aims to establish an ASEAN socio-cultural community that allows people to feel the benefits of being a part of ASEAN, and is inclusive, sustainable, resilient, and dynamic. Besides, *The Narrative of ASEAN Identity* was adopted at the 37^th^ ASEAN Summit in November 2020, reaffirming that ASEAN Identity is a process of social construct defined by balanced combination of “Constructed Values” and “Inherited Values”, which will strengthen the ASEAN Community.

## Conclusion

Despite a mounting interest in the language policies of ASEAN, the virtual language landscape of ASEAN’s official websites has often been overlooked. This research marks a pioneering endeavor by synthesizing historical web page analysis through the *Wayback Machine* tool with a corpus-based examination of the Chairman’s Statements of the ASEAN Summit (CSAS). Our investigation has revealed several pivotal insights: 1) we identified three distinct evolutionary stages of the ASEAN official website, highlighting the construction of an ASEAN community, the emphasis on cooperation, and the pursuit of regional integration; 2) we observed ASEAN’s unwavering focus on the collective “we,” underscoring the importance of collaboration and development across economic and political-security spheres; 3) our analysis showed that the ASEAN community has consistently upheld its common values throughout its development, fostering a sense of unity and ultimately constructing an ASEAN identity and a genuine ASEAN community. Overall, the current study may serve as a valuable resource for researchers, policymakers, and practitioners interested in understanding and contributing to the ongoing evolution of language policy in international or regional organizations.

However, despite these significant findings, it is crucial to recognize the inherent limitations of the study and acknowledge the necessity for further exploration. First, the database for analysis should be further expanded to include a wider range of statements or documents published on the ASEAN official website. Additionally, conducting interviews with ASEAN authorities could enhance our understanding of this topic in subsequent research. Future research is anticipated to adopt a comparative study of the language practices of member countries of ASEAN, aiming to provide a more comprehensive and inclusive analysis. Each ASEAN member possesses unique characteristics, and these differences within members may influence the language practices of ASEAN. By incorporating factors of diversity, future research will enhance our understanding of language practices and offer a more thorough comprehension of ASEAN.
